# Diagnostic Performance of Magnetic Resonance Elastography Versus Vibration‐Controlled Transient Elastography for Liver Fibrosis Staging in Steatotic Liver Disease

**DOI:** 10.1002/nbm.70429

**Published:** 2026-11-03

**Authors:** Leon David Gruenewald, Christian Booz, Jennifer Gotta, Philipp Reschke, Tommaso D'Angelo, Nicole Suarez Ziegengeist, Katerina Torgashov, Laura Hempfling, Scherwin Mahmoudi, Lisa Reisbeck, Renate Maria Hammerstingl, Stefan Zeuzem, Esra Görgülü, Anita Pathil‐Warth, Melis Onay, Eva Herrmann, Iulia Dahmer, Raja Idris, Maria Johanna Gobertina Tetuanui Vehreschild, Katharina Schregel, Christof Matthias Sommer, Sandra Ciesek, Katrin Eichler, Thomas Joseph Vogl, Ralph Sinkus, Sebastian Haberkorn, Vitali Koch

**Affiliations:** ^1^ Goethe University Frankfurt, University Hospital Department of Radiology and Nuclear Medicine Frankfurt am Main Germany; ^2^ Goethe University Frankfurt, University Hospital Department of Pediatrics Frankfurt am Main Germany; ^3^ Goethe University Frankfurt, University Hospital Department of Internal Medicine I Frankfurt am Main Germany; ^4^ Goethe University Frankfurt, Faculty of Medicine Institute of Biostatistics and Mathematical Modeling Frankfurt am Main Germany; ^5^ Goethe University Frankfurt, University Hospital Department of Internal Medicine, Infectious Diseases Frankfurt am Main Germany; ^6^ University Hospital Jena, Institute of Diagnostic and Interventional Radiology, Section Neuroradiology Jena Germany; ^7^ Goethe University Frankfurt, University Hospital, Institute of Medical Virology Frankfurt am Main Germany; ^8^ Université de Paris, Center for Research on Inflammation, UMR 1149 National Institute for Health and Medical Research (INSERM), Team “From micro to macro in cancer development” Paris France; ^9^ King's College London School of Biomedical Engineering and Imaging Sciences London UK; ^10^ Goethe University Frankfurt, University Hospital Department of Cardiology and Angiology Frankfurt am Main Germany

**Keywords:** gravitational transducer, magnetic resonance elastography, magnetic resonance imaging, metabolic dysfunction‐associated steatohepatitis, transient elastography

## Abstract

Gravitational magnetic resonance elastography (GT‐MRE) enables comprehensive biomechanical liver characterization and may overcome limitations of conventional elastography techniques. This study evaluated the diagnostic performance of two‐ and three‐dimensional (2D and 3D) GT‐MRE compared with 2D acoustic MRE (AC‐MRE) and vibration‐controlled transient elastography (VCTE) for liver fibrosis staging. In this prospective study, patients with suspected or known steatotic liver disease underwent GT‐MRE (2D and 3D, gradient‐echo sequences) and AC‐MRE (2D, spin‐echo echo‐planar imaging) at 1.5 T, plus VCTE with a median interval to MRE of 37 days (IQR, 20–124 days; range, 0–372 days). Statistical analyses included generalized additive models, ordinal logistic regression, and decision tree‐based classification. Diagnostic performance was assessed using cumulative receiver operating characteristic analysis and corresponding areas under the curve (AUCs). Ninety‐one participants (median age, 55 years [IQR, 45–64]; 41 women) were included, of whom 35 (38%) had histopathological correlation. In the biopsy‐confirmed subgroup, 3D GT‐MRE‐derived viscoelastic parameters showed promising exploratory performance for early fibrosis classification. Wave attenuation achieved AUCs of 0.91 for F0 versus ≥ F1 and 0.99 for ≤ F1 versus ≥ F2, while phase angle yielded an AUC of 0.88 for F0 versus ≥ F1, with numerically higher AUCs than conventional stiffness‐based metrics. For advanced fibrosis thresholds, all modalities performed well (AUC ≥ 0.84). Obuchowski measures for 3D‐derived parameters were 0.95–0.96. Internally cross‐validated multiparametric modeling achieved an accuracy of up to 92%. VCTE measurements plateaued beyond F3, whereas AC‐ and GT‐MRE metrics continued to increase with fibrosis severity. 3D GT‐MRE‐derived viscoelastic parameters showed promising performance for liver fibrosis classification and provided biomechanical information complementary to conventional stiffness measurements. Given the limited biopsy‐confirmed sample and marked class imbalance at F0, these findings require validation in larger independent cohorts before clinical implementation.

## Graphical abstract

In 91 patients, vibration‐controlled transient elastography, two‐dimensional acoustic magnetic resonance elastography, and two‐ and three‐dimensional gravitational magnetic resonance elastography were compared for liver fibrosis assessment, with 35 biopsy‐confirmed cases. Two‐dimensional gravitational magnetic resonance elastography showed the numerically highest area under the curve for low‐ versus high‐grade fibrosis, while three‐dimensional viscoelastic parameters showed promising early‐fibrosis discrimination. Multiparametric three‐dimensional magnetic resonance elastography achieved up to 92% internally cross‐validated accuracy.

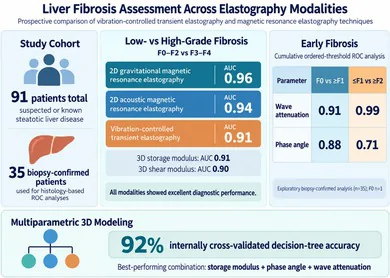

**Abbreviations**
AUC: area under the curve
GT: gravitational transducer
IQR: interquartile range
MASH: metabolic dysfunction–associated steatohepatitis
MASLD: metabolic dysfunction–associated steatotic liver disease
MRE: magnetic resonance elastography
MRI: magnetic resonance imaging
ROC: receiver operating characteristic
ROI: region of interest
SD: standard deviation
VCTE: vibration–controlled transient elastography

## 1 Introduction

Over the past three decades, the incidence and prevalence of metabolic dysfunction‐associated steatotic liver disease (MASLD) have risen sharply, establishing it as the predominant cause of end‐stage liver disease worldwide [1, 2]. Societal and dietary shifts contribute to this trend and are projected to sustain, if not increase, disease prevalence, with implications for the management of patients at risk [3]. While early‐stage MASLD often remains asymptomatic, disease progression from simple steatosis to metabolic dysfunction‐associated steatohepatitis (MASH) can ultimately advance to cirrhosis and hepatocellular carcinoma [4, 5, 6]. The progressive nature of MASLD, coupled with a lack of effective treatment strategies, underscores the importance of screening strategies at an early stage, where lifestyle and dietary modifications have the greatest protective impact [7, 8].

Liver biopsy remains the gold standard for MASH evaluation and staging; however, its routine implementation for screening purposes is precluded by inherent procedural risks, substantial costs, and sampling variability [9, 10]. Consequently, with the increasing prevalence of MASLD and the emergence of therapies targeting early fibrosis, noninvasive methods for screening, monitoring progression, and assessing treatment have gained clinical prominence. These include laboratory‐based scoring systems and imaging methods [11, 12, 13].

Ultrasound‐based vibration‐controlled transient elastography (VCTE) and magnetic resonance elastography (MRE) represent the primary noninvasive imaging modalities for hepatic fibrosis assessment [14]. Although VCTE offers rapid evaluation and seamless clinical integration, its diagnostic accuracy can be compromised in patients with obesity or ascites, is highly operator‐dependent, and exhibits substantial sampling variability. In contrast, MRE, despite reduced accessibility and higher costs, provides superior diagnostic accuracy for fibrosis detection and staging through comprehensive sampling volumes and enhanced tissue penetration depth, enabling the identification of focal heterogeneities that conventional biopsy or VCTE may fail to detect [14, 15, 16, 17, 18, 19, 20]. Conventional clinical MRE systems frequently employ acoustic (AC) transducers, typically operating at ~60 Hz, to generate shear waves, which are captured using motion‐sensitive MR sequences [21, 22]. Although widely established, AC transducers are inherently nonlinear and sensitive to preload conditions, which may result in reduced vibration amplitude or wave distortion due to higher harmonic generation, thereby limiting penetration depth and wave quality [23]. The gravitational (GT) transducer represents a novel excitation concept based on an eccentrically rotating mass [24, 25]. This design enables stable vibration amplitudes across a broad frequency range and is largely independent of preload, facilitating deeper tissue penetration and minimizing harmonic artifacts. These properties may improve wave‐field fidelity and the accuracy of viscoelastic reconstructions [25]. To date, GT‐MRE has been evaluated in 170 participants with suspected or confirmed liver disease, offering enhanced capabilities for comprehensive liver stiffness assessment and precise differentiation of fibrosis stages [24].

This study aimed to systematically compare the diagnostic performance of VCTE, two‐dimensional (2D) AC‐MRE, 2D GT‐MRE, and three‐dimensional (3D) GT‐MRE for liver fibrosis assessment. In particular, we investigated whether the additional viscoelastic parameters derived from 3D GT‐MRE provide complementary diagnostic information beyond conventional stiffness‐based measurements. By evaluating these techniques within the same patient cohort, we sought to further characterize their potential for noninvasive fibrosis staging, while recognizing that the present study was not designed to isolate the independent effects of transducer type, acquisition dimensionality, and reconstruction methodology.

## 2 Materials and Methods

The institutional ethics review board approved this prospective study (Ethics Committee of Frankfurt am Main, Hesse, Germany; approval number 20‐887_2). Written informed consent was obtained from all participants.

### 2.1 Study Population

In this prospective single‐center study, all participants aged 18 years or older with suspected or known MASLD and MASH were recruited at the University Hospital Frankfurt (Frankfurt am Main, Hesse, Germany) between November 2022 and September 2023. Exclusion criteria were the inability to undergo MRI (e.g., claustrophobia, metallic implants, or obesity exceeding the bore of the scanner), known liver disease other than MASH, substantial alcohol consumption (> 14 UK units per week for women and > 21 UK units per week for men; 1 UK unit = 10 mL of pure alcohol), hepatotoxic medication, or pregnancy. The reference standard for diagnosis was based on either histopathological confirmation or a final adjudicated clinical diagnosis when biopsy was not available. Histopathological diagnosis was considered the definitive reference standard and was used whenever liver biopsy data were available. The pathological review was conducted at the Institute of Pathology at the University Hospital Frankfurt (Frankfurt am Main, Hesse, Germany). In patients without biopsy, the diagnosis was established through a standardized clinical adjudication process incorporating clinical presentation, laboratory findings, imaging results, and longitudinal follow‐up data, in accordance with current clinical guidelines and expert consensus [8]. Importantly, VCTE, 2D AC‐MRE, 2D GT‐MRE, and 3D GT‐MRE quantitative measurements evaluated as index tests in the present study were excluded from the adjudication process. The adjudicators were blinded to these study‐specific elastography results. Clinical diagnoses were adjudicated by two experienced clinicians (L.D.G. and V.K.) who were blinded to the study‐specific imaging readouts under investigation. Of the 91 participants included in the study, 35 had histopathological fibrosis staging and 56 did not undergo liver biopsy. The full cohort was used for demographic and descriptive comparisons between participants with and without biopsy. Analyses requiring an ordinal fibrosis‐stage reference standard, including stage‐specific ROC analyses, Obuchowski measures, ordinal regression, and fibrosis‐stage classification models, were restricted to the biopsy‐confirmed subgroup (*n* = 35). The non‐biopsy subgroup was not used to determine histopathology‐referenced fibrosis‐stage diagnostic performance. Fibrosis‐stage diagnostic‐performance analyses were deliberately restricted to patients with histopathological staging to provide a uniform reference standard and to avoid potential misclassification or incorporation bias associated with imaging‐informed clinical adjudication.

### 2.2 MRE Protocol

MRE was performed on a 1.5 T MR Scanner (Magnetom Aera, Siemens Healthineers, Forchheim, Germany). The examination began with the standard MRE protocol using the AC transducer (2D acquisition; Resoundant, Rochester, Minnesota, USA), followed by dedicated 2D and 3D acquisitions using the GT transducer. The routine liver MR protocol included T2‐weighted coronal and axial sequences, as well as diffusion‐weighted imaging. The AC‐MRE sequence was a motion‐sensitive 2D spin‐echo (SE) echo‐planar imaging (EPI) sequence (TR = 1000 ms, TE = 46 ms, FOV = 380 mm, matrix size 100 × 100, and five slices) acquired during an 11‐s breath‐hold. For this part of the protocol, the AC transducer was placed over the right hepatic lobe using the xiphoid process and right midclavicular line as landmarks, transmitting 60 Hz vibrations through the upper abdomen. After completing the AC‐MRE protocol, the GT transducer was positioned at the same height in the right midaxillary line to align its primary vibration direction with the readout direction (right–left), minimizing motion‐related artifacts. Both 2D and 3D acquisitions used the Ristretto scheme with fractional motion encoding and a fixed excitation frequency of 60 Hz [26]. Imaging parameters were TE/TR = 9.53 ms/54 ms for 2D and 9.53 ms/104.25 ms for 3D. The 2D sequence covered four 8 mm transverse slices, whereas the 3D sequence covered eight 4 mm slices; the in‐plane resolution was 4 mm isotropic for both sequences. A GRAPPA (GeneRalized autocalibrating partial parallel acquisition) factor of 2 was applied, resulting in breath‐hold durations of 13 s for 2D and 4 × 14 s for 3D. The GT‐MRE protocol was completed in approximately 150 s. Total acquisition time for the product MRE protocol was similar, as both gradient‐echo (GRE)‐based and SE‐EPI MRE sequences were acquired. Signal reception utilized the 18‐channel body matrix coil, along with elements of the integrated 32‐channel spine matrix coil. The GT‐MRE research demonstrator and hardware setup have been described previously [24]. A detailed description of MRE parameters is provided in Table S1.

### 2.3 Method for GT 3D and 2D Reconstruction of Stiffness

The GT 3D‐MRE reconstruction method used in this study has previously been described in detail [27]. Briefly, the application of the mathematical curl operator removes contributions from the compressional wave, which cannot be reliably processed because of its substantially higher propagation speed compared with the shear wave (approximately 1550 m/s vs. 1–10 m/s, respectively). The resulting Helmholtz equation allows for solving of the complex‐valued shear modulus $\left|G^{*}\right|$, i.e., for elasticity $G^{\prime }$ and viscosity $G^{\prime \prime }$ in a minimum $\chi^{2}$‐manner for the square of the wave‐vector ${\vec{k}}^{2}=\frac{\rho \omega^{2}}{G^{*}}$, where ρ is the tissue density (assumed to be constant and equal to that of water) and ω is the angular frequency of the mechanical vibration. However, here, only its magnitude $\left|G^{*}\right|=\sqrt{G\prime^{2}+G\prime \prime^{2}}$ is compared across different methods. The phase angle was defined as $Y=\frac{2}{\pi }\tan^{-1}\left(\frac{G^{\prime \prime }}{G^{\prime }}\right),$ corresponding to the phase of the complex shear modulus normalized to π/2. Wave attenuation was derived from the imaginary component of the complex wave number as α=Imρω2/G* and is reported in m^−1^. For the GT 2D approach, we used the directional filter method, which (i) suppresses compressional wave components via high‐pass filtering, since the curl cannot be computed in 2D, and (ii) simplifies inversion of the wave equation by isolating specific directional components of the displacement field using a pie‐chart selection in Fourier space. Equivalent maps (i.e., magnitude of the complex shear modulus) were created using our proposed 2D/3D reconstruction algorithms [24]. Confidence and stiffness maps ($\left|G^{*}\right|$) for the AC 2D results were created with the standard product inversion algorithm (“MMDI v3.0.4”, Resoundant, Rochester, Minnesota, USA).

### 2.4 Creation of Region of Interests (ROIs) for Analysis

A radiologist with 7 years of experience in abdominal imaging (L.D.G.) and a biomedical engineer with 22 years of experience in elastography and 12 years of experience in liver imaging (R.S.) independently performed ROI placement. Readers were blinded to histopathological fibrosis stage, clinical and laboratory information, VCTE results, and quantitative results from the other MRE techniques. Modality‐specific ROIs were independently drawn for 2D AC‐MRE, 2D GT‐MRE, and 3D GT‐MRE and were not transferred or co‐registered between acquisitions. Anatomical images and modality‐specific confidence/quality maps were available to the readers because these were required for appropriate ROI placement to avoid inclusion of extrahepatic structures. ROIs were designed to cover the outer liver parenchyma while excluding large vessels in deeper regions. Pixels within the ROIs that failed quality checks were excluded from the stiffness calculations. To ensure data integrity, datasets were discarded if they did not meet a minimum requirement for the number of valid pixels within the abdomen and within the ROI used for stiffness quantification. Quantitative elasticity values were obtained separately from ROIs drawn by Reader 1 and Reader 2. Although two independent readers set and assessed the ROIs, two additional radiologists with at least 3 years of experience in abdominal imaging (J.G. and C.B.) evaluated all MRE datasets.

### 2.5 VCTE Image Acquisition

Liver stiffness measurement (LSM) was performed using VCTE and controlled attenuation parameter (CAP) assessment with FibroScan 630 Touch device equipped with both M and XL probes (Echosens, Paris, France). All examinations were conducted by physicians trained and certified by the manufacturer. Investigators were blinded to histological results. Patients fasted for at least 3 h before the exam. Probe type was automatically selected using the device's integrated probe selection tool. With patients in the supine position and the right arm fully abducted, measurements were obtained from the right liver lobe through an intercostal space. CAP represents the mean ultrasound attenuation at 3.5 MHz and is reported in dB/m. LSM by VCTE reflects the average tissue stiffness (Young's modulus) at a shear wave frequency of 50 Hz and is expressed in kPa. Only studies with at least 10 valid measurements were considered acceptable. VCTE was performed within a median of 37 days (IQR, 20–124 days, range 0–372 days).

### 2.6 Statistical Analysis

All statistical analyses were performed using R (version 4.2.2; R Foundation for Statistical Computing, Vienna, Austria), GraphPad Prism (version 10.0; GraphPad Software, San Diego, California, USA), and Python (version 3.11; Python Software Foundation, Wilmington, Delaware, USA). Continuous variables were tested for normality using the Shapiro–Wilk test. Variables following a normal distribution were expressed as mean ± standard deviation and compared between groups using Student's *t*‐tests. Non‐normally distributed variables were reported as medians and interquartile ranges (IQRs) and analyzed using the Mann–Whitney *U* test. Categorical variables were summarized as frequencies and percentages and compared using chi‐squared tests with continuity correction for 2 × 2 comparisons, as appropriate. Receiver operating characteristic (ROC) analysis was used to evaluate diagnostic performance. Cumulative ROC curves were constructed to assess the discrimination of ordered fibrosis stages (e.g., ≥ F1, ≥ F2, etc.) using optimized thresholds. Additionally, the diagnostic performance using established clinical stiffness cutoffs was evaluated. To quantify global diagnostic accuracy across the full ordinal spectrum, the Obuchowski Measure (OM) was calculated. Area under the curve (AUC), sensitivity, specificity, positive predictive value (PPV), negative predictive value (NPV), and overall accuracy were reported. To assess the relationship between imaging parameters and fibrosis classification, several modeling approaches were applied. Ordinal logistic regression was used to estimate the predictive value of individual and combined metrics, and model performance was quantified using McFadden's pseudo‐R [2]. Generalized additive models (GAMs) were employed to capture potential nonlinear associations between imaging values and fibrosis stage; model fit was assessed using deviance explained. Additionally, decision tree models with a maximum depth of 3 were trained to evaluate the classification accuracy of both individual parameters and combinations. Combinations of 3D‐MRE‐derived metrics‐such as shear modulus, wave attenuation, phase angle, and shear wave speed were included in comparative model evaluations. Interreader agreement for region‐of‐interest (ROI) measurements was evaluated using weighted Cohen's kappa statistics. This approach accounts for the degree of disagreement between readers by assigning greater weight to larger discrepancies, thereby providing a more nuanced assessment than unweighted kappa. Kappa values were interpreted according to established criteria, with values indicating slight (≤ 0.20), fair (0.21–0.40), moderate (0.41–0.60), substantial (0.61–0.80), and excellent agreement (> 0.80). Corresponding 95% confidence intervals were calculated to quantify the precision of agreement estimates. Given the limited number of biopsy‐confirmed cases and the application of multiple statistical models, *p*‐values were adjusted for multiple comparisons using the Benjamini–Hochberg false discovery rate (FDR) correction, with an FDR threshold of 5%. To mitigate overfitting in the small biopsy‐confirmed cohort, decision tree complexity was constrained to a maximum depth of three. Model performance was assessed using repeated k‐fold cross‐validation, with accuracy reported as the mean across validation folds. Owing to the limited sample size, no independent test set was used, and performance metrics should therefore be interpreted as internally validated and exploratory. A *p*‐value < 0.05 was considered statistically significant.

## 3 Results

### 3.1 Patient Characteristics

A total of 91 patients (median age 55 years (IQR, 45–64); 50 males [55%], 41 females [45%]) were enrolled, of whom 35 (38%) underwent liver biopsy. Biopsied patients were older than those without biopsy (59 years [IQR, 50–68] vs. 52 years [IQR, 43–61]; *p* = 0.04). Sex distribution did not differ between groups (male: 49% vs. 59%; female: 51% vs. 41%, *p* = 0.45). Metabolic risk factors also showed a similar distribution. Median body mass index (BMI) was 28.1 kg/m^2^ (IQR, 25.9–31.0) overall and did not differ significantly between the biopsy and non‐biopsy groups (27.2 [IQR, 25.8–31.0] vs. 28.3 [IQR, 26.7–30.9] kg/m^2^; *p* = 0.41). The prevalence of obesity reached 83% and 84% in both cohorts, respectively (*p* = 1.00). Alcohol consumption (17% vs. 14%, *p* = 0.95), diabetes (31% vs. 34%, *p* = 0.99), and hypercholesterolemia (83% vs. 88%, *p* = 0.76) rates showed no statistically significant differences. Regarding the metabolic liver disease classification, MASLD was identified in 13% of the total patient population, with no significant difference between the groups (9% in the biopsy cohort vs. 16% in the non‐biopsy cohort; *p* = 0.48). The majority of patients received a classification of metabolic dysfunction‐associated steatohepatitis (MASH), with a nonsignificant trend toward higher prevalence in the biopsy group (86% vs. 71%; *p* = 0.19). Detailed patient characteristics are presented in Table 1. The study population is illustrated in Figure 1.

**TABLE 1:** Baseline patient characteristics.

Variables	Total (*n* = 91)	Biopsy (*n* = 35)	No biopsy (*n* = 56)	*p*
Age (years), median (IQR)	55 (45–64)	59 (50–68)	52 (43–61)	0.04
Sex				
Male (*n* [%])	50 (55%)	17 (49%)	33 (59%)	0.45
Female (*n* [%])	41 (45%)	18 (51%)	23 (41%)	
Metabolic risk factors				
BMI (kg/m^2^), median (IQR)	28.1 (25.9–31.0)	27.2 (25.8–31.0)	28.3 (26.7–30.9)	0.41
Obesity (*n* [%])	76 (84%)	29 (83%)	47 (84%)	1.00
Alcohol consumption (*n* [%])	14 (15%)	6 (17%)	8 (14%)	0.95
Diabetes (*n* [%])	30 (33%)	11 (31%)	19 (34%)	0.99
Hypercholesterolemia (*n* [%])	78 (86%)	29 (83%)	49 (88%)	0.76
Metabolic characterization				
MASLD (*n* [%])	12 (13%)	3 (9%)	9 (16%)	0.48
MASH (*n* [%])	70 (77%)	30 (86%)	40 (71%)	0.19

*Note:* Continuous variables were tested for normality using the Shapiro–Wilk test. Non‐normally distributed variables are presented as median (interquartile range) and were compared using the Mann–Whitney *U* test. Categorical variables are presented as *n* (%) and were compared using chi‐square tests with continuity correction, as appropriate.

Abbreviations: BMI, body mass index; IQR, interquartile range; MASH, metabolic dysfunction‐associated steatohepatitis; MASLD, metabolic dysfunction‐associated steatotic liver disease; SD, standard deviation.

**FIGURE 1:**
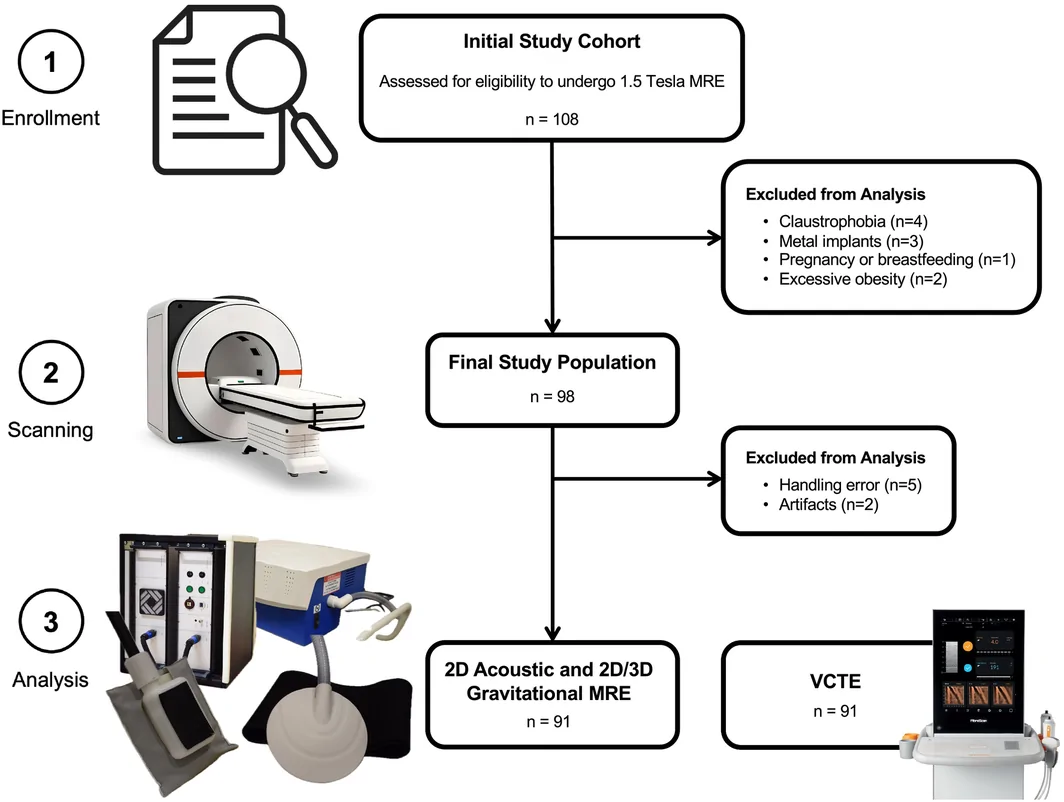
Characteristics of the study population. Abbreviations: MRE, magnetic resonance elastography; VCTE, vibration‐controlled transient elastography.

### 3.2 Baseline Metrics Across Elastography Modalities

VCTE measurements were similar between the biopsy and non‐biopsy groups (7.60 kPa [IQR, 5.3–12.2] vs. 6.25 kPa [IQR, 4.3–9.9]; *p* = 0.10). AC‐MRE showed higher stiffness in the biopsy group (3.22 kPa [IQR, 2.2–5.2] vs. 2.50 kPa [IQR, 2.1–3.3]; *p* = 0.04), whereas 2D GT‐MRE showed no significant difference between both groups (3.16 kPa [IQR, 2.2–4.8] vs. 2.67 kPa [IQR, 2.2–3.5]; *p* = 0.27). Among 3D GT‐MRE parameters, differences between groups were not statistically significant for shear modulus (3.26 kPa [IQR, 2.0–5.4] vs. 2.33 kPa [IQR, 1.9–3.2]; *p* = 0.051), storage modulus (2.86 kPa [IQR, 1.8–4.3] vs. 2.06 kPa [IQR, 1.6–2.9]; *p* = 0.06), or loss modulus (1.03 kPa [IQR, 0.8–1.9] vs. 0.92 kPa [IQR, 0.8–1.1]; *p* = 0.06). Similarly, no significant differences were identified for the maximum shear wave speed (1.84 m/s [IQR, 1.4–2.4] vs. 1.56 m/s [IQR, 1.4–1.8]; *p* = 0.06) or the wave attenuation (43.65 [IQR, 35.8–58.4] vs. 50.20 [IQR, 42.2–58.2]; *p* = 0.10). Interreader agreement for ROI measurements was excellent (weighted κ = 0.93, 95% CI: 0.89–0.97). Across all fibrosis stages, no significant association was observed between fibrosis grade and either histological steatosis grade or quantitative fat fraction. Specifically, the mean steatosis grade was 1.0 for fibrosis stage F0, 1.57 ± 0.85 for F1, 1.57 ± 0.53 for F2, 1.10 ± 0.57 for F3, and 1.50 ± 0.58 for F4. One‐way analysis of variance demonstrated no significant difference in steatosis grade across fibrosis stages (*p* = 0.49). Similarly, quantitative fat fraction did not differ significantly between fibrosis grades. Mean fat fraction was 5.3% for F0, 13.8% ± 8.7% for F1, 11.9% ± 6.5% for F2, 13.1% ± 7.3% for F3, and 16.5% ± 9.5% for F4. No statistically significant difference in fat fraction across fibrosis stages was observed (*p* = 0.75). Detailed liver stiffness metrics are given in Table 2.

**TABLE 2:** Baseline liver stiffness measurements across imaging modalities.

Variables	Total (*n* = 91)	Biopsy (*n* = 35)	No biopsy (*n* = 56)	*p*
VCTE (kPa)	6.60 (4.4–11.5)	7.60 (5.3–12.2)	6.25 (4.3–9.9)	0.10
2D AC‐MRE (kPa)	2.75 (2.1–4.0)	3.22 (2.2–5.2)	2.50 (2.1–3.3)	0.04
2D GT‐MRE (kPa)	2.72 (2.2–4.0)	3.16 (2.2–4.8)	2.67 (2.2–3.5)	0.27
3D GT‐MRE				
Shear modulus (kPa)	2.38 (1.9–3.8)	3.26 (2.0–5.4)	2.33 (1.9–3.2)	0.051
Storage modulus (kPa)	2.12 (1.7–3.5)	2.86 (1.8–4.3)	2.06 (1.6–2.9)	0.06
Loss modulus (kPa)	0.93 (0.8–1.4)	1.03 (0.8–1.9)	0.92 (0.8–1.1)	0.06
Shear wave speed (m/s)	1.57 (1.4–2.0)	1.84 (1.4–2.4)	1.56 (1.4–1.8)	0.06
Wave attenuation (1/m)	50.09 (37.9–58.5)	43.65 (35.8–58.4)	50.20 (42.2–58.2)	0.10

*Note:* Comprehensive liver stiffness metrics obtained from transient elastography (VCTE), AC‐MRE, and 2D/3D GT‐MRE. Data are presented as median (interquartile range) following rejection of normality. Between‐group comparisons employed the Mann–Whitney *U* test.

Abbreviations: AC, acoustic; GT, gravitational; MRE, magnetic resonance elastography; VCTE, vibration‐controlled transient elastography.

### 3.3 Generalized Additive Models for Stiffness Relationship Analysis

The implementation of GAMs enabled a comprehensive assessment of the relationships between MRE‐derived stiffness metrics and VCTE values. Both the 2D AC‐ and 2D GT‐MRE demonstrated approximately linear relationships with VCTE measurements up to ~18 kPa ultrasound stiffness, beyond which the VCTE values plateaued whereas the MRE values continued to increase. Similar patterns emerged across the 3D GT parameters, with linear relationships maintained up to ~18–22 kPa, followed by VCTE value plateauing or declining. 2D AC‐MRE achieved the highest deviance explained (63.6%, *p* < 0.001), followed by the 3D shear modulus (51.9%, *p* < 0.001) and the maximum shear wave speed (53.4%, *p* < 0.001). These findings indicate fundamentally divergent stiffness response characteristics between the modalities at higher levels of fibrosis (Figure 2) (Table 3).

**FIGURE 2:**
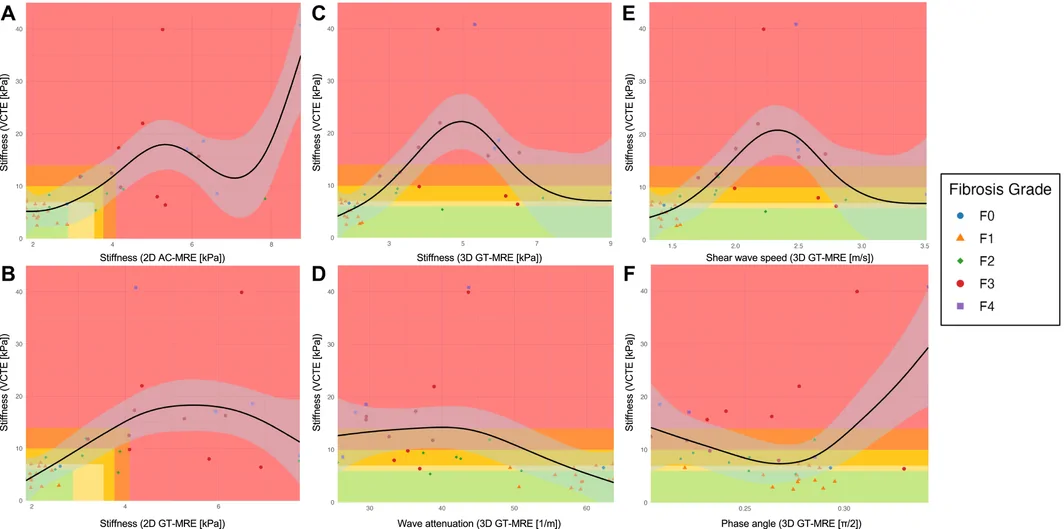
Generalized additive models demonstrating the relationship between VCTE‐derived stiffness (kPa) and MRE‐derived metrics across 2D AC‐MRE, 2D GT‐MRE, and 3D GT‐MRE modalities. Comprehensive visualization of generalized additive model (GAM) plots illustrating the modeled associations between MRE‐derived stiffness values (x‐axis) and VCTE measurements (y‐axis) in the cohort of patients with histologically confirmed fibrosis (*n* = 35). 2D AC‐ and 2D GT‐MRE demonstrate an approximately linear correlation with VCTE measurements up to ~18 kPa. Beyond this range, VCTE values tend to plateau, whereas MRE values continue to increase progressively. Similar behavioral patterns are observed for 3D parameters, with linear relationships maintained until 18–22 kPa, after which the VCTE value plateaus. The shaded areas represent 95% confidence intervals for the modeled relationships. The colored bands delineate established fibrosis stage cutoff thresholds: F0 (green), F1 (light yellow), F2 (dark yellow), F3 (orange), and F4 (red). Abbreviations: AC, acoustic; GAM, generalized additive model; GT, gravitational; kPa, kilopascals; MRE, magnetic resonance elastography; VCTE, vibration‐controlled transient elastography.

**TABLE 3:** Relationship between MRE‐derived metrics and VCTE Stiffness in generalized additive models.

Parameter	Estimated degrees of freedom	Deviance explained	*p*
2D AC‐MRE (kPa)	5.6	63.6%	< 0.001
2D GT‐MRE (kPa)	2.4	40.1%	0.001
3D GT‐MRE			
Shear modulus (kPa)	4.2	51.9%	< 0.001
Storage modulus (kPa)	2.3	44.2%	0.001
Loss modulus (kPa)	2.4	37.8%	0.001
Shear wave speed (m/s)	4.3	53.4%	< 0.001
Phase angle (π/2)	2.4	32.5%	0.01

*Note:* Generalized additive models enable assessment of non‐linear relationships between MRE‐derived parameters and transient elastography values in the biopsy‐confirmed subgroup (*n* = 35). Higher deviance explained indicates superior model fit to the VCTE stiffness distribution. Estimated degrees of freedom values exceeding 1.0 indicate the presence of non‐linearity in the modeled relationship.

Abbreviations: AC, acoustic; GAM, generalized additive model; GT, gravitational; MRE, magnetic resonance elastography; VCTE, vibration‐controlled transient elastography.

### 3.4 Fibrosis Classification in Biopsy‐Confirmed Subset

Within the cohort of 35 patients with histologically confirmed fibrosis, the classification distribution demonstrated substantial variability between modalities when applying established thresholds [20, 28]. Histopathological assessment revealed the highest proportion of patients in stage F1, while VCTE demonstrated a more uniform distribution across all fibrosis stages. Both MRE‐based techniques exhibited clustering of patients predominantly in stages F0 and F4, with a markedly smaller number of patients classified into the intermediate fibrosis stages. Full classification counts for each modality are shown in Table 4.

**TABLE 4:** Comparison of fibrosis stage classification by imaging modality versus histopathology in patients with liver biopsy.

Grading and acquisition method	0	1	2	3	4
Histopathology (*n* [%])	1 (3%)	13 (37%)	7 (20%)	10 (29%)	4 (11%)
VCTE (*n* [%])	11 (31%)	5 (14%)	8 (23%)	3 (9%)	8 (23%)
2D AC‐MRE (*n* [%])	16 (46%)	2 (6%)	1 (3%)	2 (6%)	14 (40%)
2D GT‐MRE (*n* [%])	16 (46%)	3 (9%)	0 (0%)	3 (9%)	13 (37%)

*Note:* Comprehensive fibrosis stage classification comparison in the cohort of 35 patients with histological confirmation. Imaging‐based classifications employed established stiffness thresholds for VCTE and MRE. Data are presented as *n* (%) of the histologically confirmed cohort. Percentages may not sum to 100% due to rounding.

Abbreviations: AC, acoustic; GT, gravitational; MRE, magnetic resonance elastography; VCTE, vibration‐controlled transient elastography.

### 3.5 ROC Analysis for Fibrosis Stage Differentiation

We performed three ROC analyses to assess the diagnostic performance of each modality: differentiation of low‐grade (F0–F2) versus high‐grade (F3–F4) fibrosis (Figure 3), precise fibrosis staging using established cutoffs (Figure 4), and cumulative stage‐threshold ROC analysis across all parameters (Figure 5).

**FIGURE 3:**
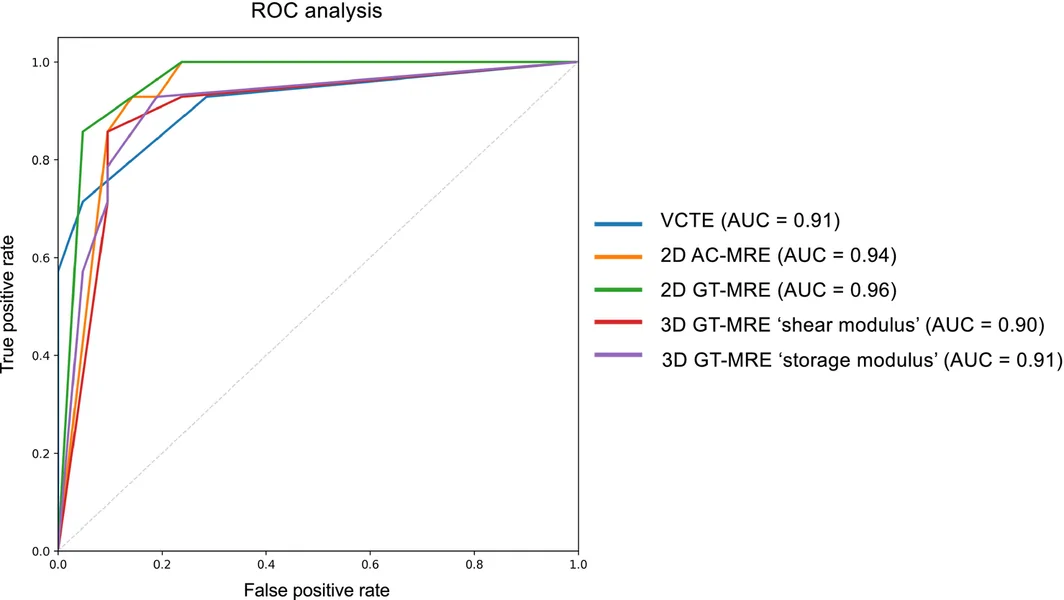
ROC curves for differentiating low‐grade (F0‐F2) versus high‐grade (F3‐F4) liver fibrosis. Receiver operating characteristic (ROC) analysis demonstrating the comparative diagnostic performance of VCTE, 2D AC‐MRE, as well as 2D & 3D GT‐MRE‐derived parameters for binary fibrosis classification using histopathological reference standard (*n* = 35). All evaluated modalities demonstrated excellent discrimination. AUC values ranged from 0.90 to 0.96. The 2D GT‐MRE measurement yielded the numerically highest AUC (0.96). Abbreviations: AC, acoustic; AUC, area under the curve; GT, gravitational; MRE, magnetic resonance elastography; ROC, receiver operating characteristic; VCTE, vibration‐controlled transient elastography.

**FIGURE 4:**
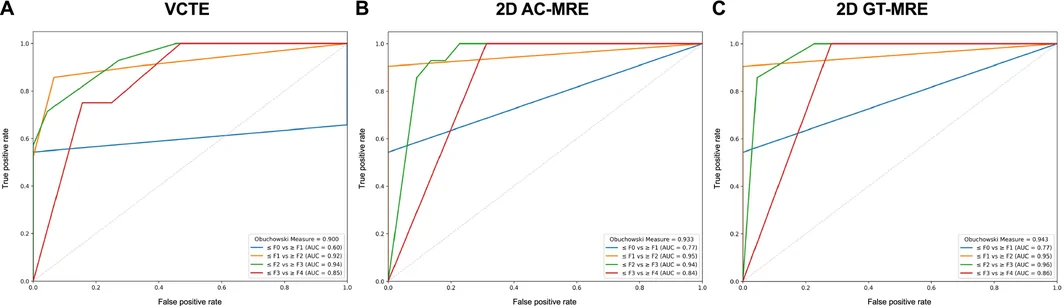
Stage‐specific ROC curves for fibrosis classification using established stiffness cutoffs. Comprehensive ROC curve analysis for precise fibrosis staging (F0 through F4) employing predefined stiffness thresholds for VCTE, 2D AC‐MRE, and 2D GT‐MRE in the cohort of 35 patients with a histological reference standard. The highest diagnostic accuracy in detecting advanced fibrosis (stage F4) was achieved with the 2D GT‐MRE system demonstrating an AUC of 0.86. VCTE exhibited limited performance in detecting early‐stage fibrosis, with F0 vs. ≥ F1 thresholds not exceeding 0.60, highlighting a critical diagnostic gap in early disease identification. Abbreviations: AC, acoustic; AUC, area under the curve; F0–F4, fibrosis stages 0–4; GT, gravitational; MRE, magnetic resonance elastography; ROC, receiver operating characteristic.

**FIGURE 5:**
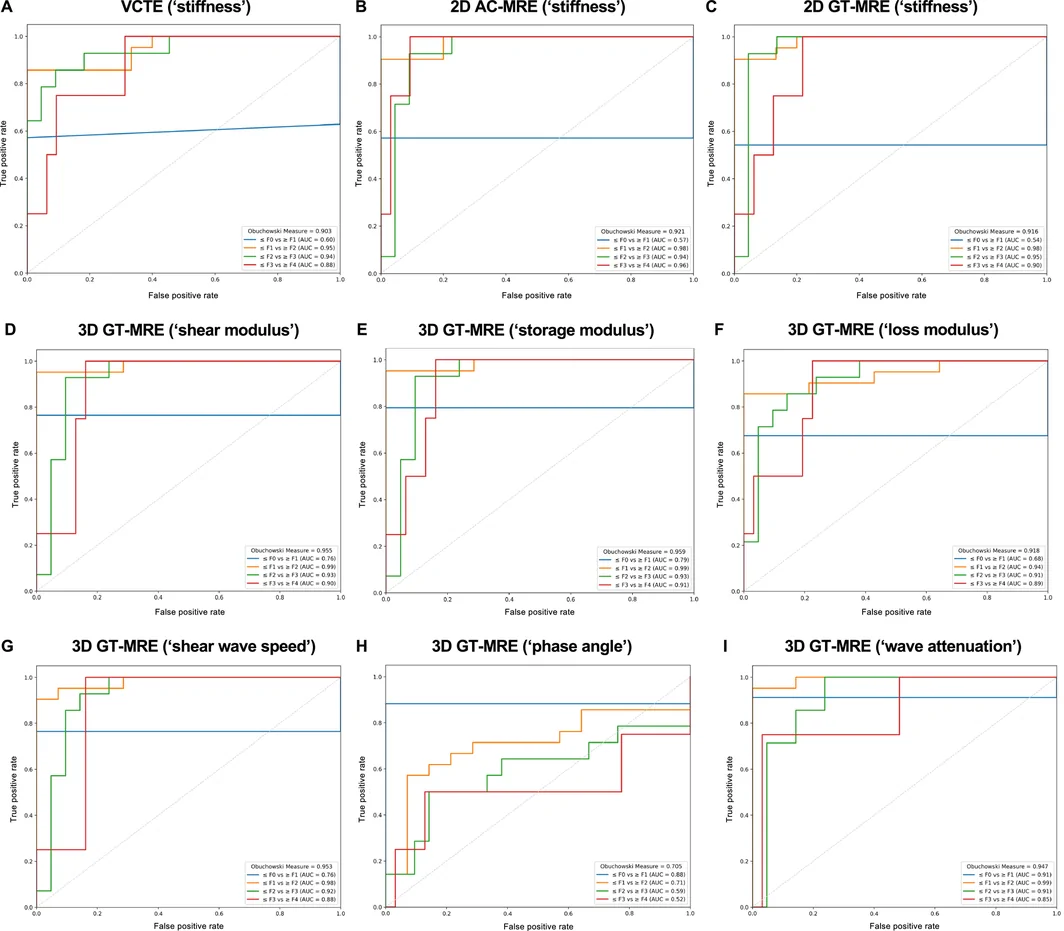
Cumulative ROC analysis across ordered fibrosis‐stage thresholds. Cumulative ROC analysis using optimized thresholds assessed discrimination across ordered fibrosis‐stage thresholds comparing the diagnostic performance of all imaging‐derived stiffness metrics for ordered fibrosis‐stage thresholds (F0 through F4) using a histopathological reference standard in 35 patients. 3D wave attenuation yielded AUC values of 0.91 for F0 versus ≥ F1 and 0.99 for ≤ F1 versus ≥ F2. Phase angle yielded an AUC of 0.88 for F0 vs. ≥ F1 and 0.71 for ≤ F1 versus ≥ F2. In marked contrast, VCTE and 2D‐MRE metrics showed lower numerical AUCs for the early fibrosis thresholds. For advanced fibrosis stages (F3–F4), multiple parameters demonstrated excellent performance. The F0 estimate is based on one histopathologically classified F0 participant and should therefore be interpreted descriptively. Abbreviations: AUC, area under the curve; MRE, magnetic resonance elastography; ROC, receiver operating characteristic; VCTE, vibration‐controlled transient elastography.

For the classification of low‐grade versus high‐grade fibrosis, all imaging modalities demonstrated excellent diagnostic performance. The AUC was numerically highest for 2D GT‐MRE (AUC = 0.96, 95% CI: 0.92–0.99; *p* < 0.001), followed by AC‐MRE (AUC = 0.94, 95% CI: 0.89–0.98; *p* < 0.001), and VCTE (AUC = 0.91, 95% CI: 0.86–0.96; *p* < 0.001). The 3D storage and shear moduli also showed strong performance (AUC = 0.91, 95% CI: 0.85–0.96 and AUC = 0.90, 95% CI: 0.84–0.95, respectively; both *p* < 0.001). Across modalities, sensitivity ranged from 0.86 to 0.93 and specificity from 0.81 to 0.95 (Figure 3). When evaluating exact fibrosis stages using established stiffness thresholds, high diagnostic accuracy was observed for advanced fibrosis (F3‐F4). 2D GT‐MRE yielded the numerically highest AUC for F4 (AUC = 0.86, 95% CI: 0.79–0.92; *p* < 0.001), with a sensitivity of 1 and a specificity of 0.72. VCTE and AC‐MRE followed with AUCs of 0.85 (95% CI: 0.77–0.92) and 0.84 (95% CI: 0.76–0.91), respectively (*p* < 0.001). In contrast, VCTE, AC‐MRE, and 2D GT‐MRE showed limited discrimination at the F0‐versus‐ ≥ F1 threshold, with AUCs ≤ 0.77 (95% CIs crossing 0.50; *p* > 0.05) (Figure 4).

Cumulative ROC analysis using optimized thresholds assessed discrimination across ordered fibrosis‐stage thresholds and revealed differences in performance between parameters (Figure 5). Although most modalities accurately identified advanced fibrosis, only 3D‐derived metrics demonstrated high diagnostic accuracy for early fibrosis. 3D wave attenuation yielded an AUC of 0.91 for F0 versus ≥ F1 (95% CI: 0.85–0.96; *p* < 0.001) and AUC of 0.99 for ≤ F1 versus ≥ F2 (95% CI: 0.96–1.00; *p* < 0.001). 3D phase angle also performed well for F0 versus ≥ F1 (AUC = 0.88, 95% CI: 0.82–0.94; *p* < 0.001) and yielded an AUC of 0.71 (95% CI: 0.59–0.83; *p* > 0.05) for ≤ F1 versus ≥ F2. Conventional stiffness‐based VCTE and 2D‐MRE showed poor discrimination for early fibrosis (AUCs ≤ 0.60). This pattern was confirmed by the global Obuchowski Measure, with higher values for 3D‐derived parameters (0.95–0.96; 95% CI: 0.92–0.98) compared with conventional stiffness metrics (0.90–0.92; 95% CI: 0.87–0.95).

### 3.6 Model‐Based Fibrosis Classification Performance

The evaluation of predictive performance employed three complementary modeling approaches: ordinal logistic regression, GAMs, and decision trees with maximum depth constrained to three levels (Table 5). Among the individual metrics evaluated, the 3D wave attenuation yielded the highest performance across all modeling approaches, achieving a pseudo‐*R* ^2^ of 0.46 in ordinal regression analysis, 47.5% deviance explained in the GAM framework, and an accuracy of 0.87 in decision tree classification (*p* < 0.001). VCTE demonstrated the lowest pseudo‐R^2^ (0.21), and deviance explained (33.3%, *p* < 0.01), with a decision tree accuracy of 0.79 (*p* > 0.05).

**TABLE 5:** Predictive performance of imaging metrics and their combinations for accurate fibrosis classification across statistical models.

Variables	Ordinal logistic regression (pseudo *R* ^2^)	Ordinal GAM (deviance explained)	Decision tree depth = 3 (accuracy)
VCTE (kPa)	0.21	33.3%	0.79
2D AC‐MRE (kPa)	0.38	42.3%	0.80
2D GT‐MRE (kPa)	0.33	44.5%	0.81
3D GT‐MRE			
Shear modulus (kPa)	0.32	55.9%	0.78
Storage modulus (kPa)	0.36	49.1%	0.78
Shear wave speed (m/s)	0.35	50.0%	0.77
Wave attenuation (1/m)	0.46	47.5%	0.87
Shear modulus + shear wave speed + phase angle	0.48	**78.4%**	0.76
Storage modulus + phase angle + wave attenuation	**0.49**	54.5%	**0.92**

*Note:* Comprehensive performance evaluation metrics for individual imaging parameters and strategically combined 3D‐MRE parameters across three distinct statistical modeling approaches. Pseudo‐R^2^ represents McFadden's pseudo R‐squared for ordinal logistic regression models. Deviance explained serves as the primary model fit metric for generalized additive models. Decision tree accuracy reflects classification performance using constrained trees with maximum depth of three levels. All models were trained in the biopsy‐confirmed subgroup (*n* = 35), using histologically confirmed fibrosis stages as the reference outcome. Bold values indicate optimal performance within each modeling approach.

Abbreviations: AC, acoustic; GAM, generalized additive model; GT, gravitational; MRE, magnetic resonance elastography; VCTE, vibration‐controlled transient elastography.

The strategic combination of 3D parameters resulted in substantial performance improvements. The combination of shear modulus, shear wave speed, and phase angle achieved the highest GAM deviance explained (78.4%; *p* < 0.001), whereas the integration of storage modulus, phase angle, and wave attenuation produced the highest decision tree accuracy (0.92; *p* < 0.001). These results suggest potential value of multiparametric modeling approaches to enhance fibrosis stage prediction beyond the capabilities of single‐metric classifiers.

## 4 Discussion

This study prospectively evaluated and compared the diagnostic performance of VCTE, 2D AC‐MRE, and 2D‐ and 3D GT‐MRE, including 3D viscoelastic parameters, for noninvasive liver fibrosis staging, with stage‐specific diagnostic analyses performed in the biopsy‐confirmed subgroup. Consistent with prior reports on the accuracy of MRE‐based techniques, we found that all three modalities demonstrated excellent ability to distinguish between low‐grade and high‐grade fibrosis. However, 3D GT‐MRE, incorporating phase angle and wave attenuation parameters, showed numerically higher AUCs than both VCTE and AC‐MRE for detecting early‐stage (F0–F1) fibrosis and provided finer discrimination across the full disease spectrum.

The observed associations of wave attenuation and phase angle with fibrosis stage suggest that these parameters may provide complementary information on liver tissue viscoelasticity beyond conventional stiffness measurements. The quantification of wave energy dissipation via wave attenuation may capture nascent collagen deposition and hepatocellular ballooning characteristic of early MASH progression [29]. The observed phase angle alterations indicate shifts in the viscous‐to‐elastic ratio of hepatic tissue, potentially reflecting the inflammatory infiltration and early stages of fibrotic remodeling [30]. However, these parameters are not histologically specific. Wave attenuation and phase angle may be influenced by multiple tissue characteristics, including fibrosis, inflammation, edema, vascular or perfusion changes, fat content, tissue heterogeneity, and acquisition or reconstruction conditions. Previous investigations have demonstrated that tissue viscoelastic properties undergo measurable changes before the accumulation of substantial collagen deposits [31]. Our findings extend these observations, suggesting that 3D volumetric assessment of wave propagation characteristics may provide complementary sensitivity to these early pathophysiological alterations compared to conventional 2D approaches or single‐frequency measurements.

Although transient elastography is widely used due to its accessibility and ease of implementation, its diagnostic performance becomes substantially compromised in patients with obesity or ascites and demonstrates significant operator dependency with considerable sampling variability [30]. MRE has consistently shown superior reproducibility and accuracy in liver stiffness measurement across diverse patient populations [20]. Our results align with these established observations and further demonstrate that GT‐MRE—particularly when leveraging 3D viscoelastic parameters—offers more comprehensive biomechanical tissue characterization and may provide additional information for stage discrimination, including the diagnostically challenging early stages.

Notably, wave attenuation yielded AUCs of 0.91 for F0 versus ≥ F1 and 0.99 for ≤ F1 versus ≥ F2, whereas phase angle yielded AUCs of 0.88 and 0.71 for the corresponding thresholds, respectively. These AUCs were generally numerically higher than those obtained using conventional stiffness‐based measurements. These observed numerical differences suggest that quantifying wave dispersion and energy dissipation, as captured by wave attenuation, may reflect microstructural tissue alterations, such as lobular inflammation or early perisinusoidal fibrotic bridging, that remain undetected by conventional stiffness measurements alone. Previous investigations have highlighted a similar diagnostic potential of viscoelastic parameters in animal models and preliminary human studies; however, our work represents one of the first exploratory biopsy‐controlled evaluations within the specific context of MASH [30, 32].

For the detection of high‐grade fibrosis (F3‐F4), all evaluated modalities demonstrated robust performance, consistent with the findings reported in existing meta‐analyses [28]. When applying established stiffness thresholds, 2D GT‐MRE yielded the highest diagnostic accuracy for cirrhosis (AUC = 0.86). However, the discriminatory power of 3D‐MRE across the entire disease spectrum has been evaluated by the global Obuchowski Measure (0.95–0.96), which consistently exceeded that of transient elastography and 2D‐MRE (0.90–0.92). Our analysis further revealed that VCTE values demonstrated a characteristic plateauing behavior beyond approximately 18 kPa, whereas the MRE measurements, particularly the 3D shear modulus and shear wave speed parameters, continued to indicate progressive scaling with increasing fibrosis severity. This plateauing phenomenon may contribute to the misclassification of severe fibrosis when using transient elastography, a limitation previously documented in MASH patient populations [33]. Our comprehensive GAM and ROC analyses suggest that MRE, particularly when operating in 3D mode, maintains measurement linearity across the entire fibrosis spectrum and provides enhanced granularity even in advanced disease states.

Our findings also highlight the added value of multi‐parametric modeling. Combining viscoelastic features (e.g., storage modulus, phase angle, and wave attenuation) improved predictive accuracy for fibrosis staging beyond single‐parameter approaches. In decision trees (depth = 3), this combination reached 92% internally cross‐validated accuracy, surpassing all individual measures. These results support emerging frameworks that advocate for composite MRE‐based scores rather than relying solely on stiffness thresholds. Moreover, such combinations may reduce variability caused by co‐existing conditions such as steatosis or active inflammation. The multiparametric approach provides complementary pathophysiological information regarding tissue biomechanical properties. Although the shear modulus primarily reflects bulk tissue stiffness, which may be influenced by fibrosis and other tissue characteristics, wave attenuation characterizes energy dissipation during wave propagation but is not specific to an individual histopathological process. The phase angle quantifies the dynamic balance between elastic (reversible) and viscous (irreversible) tissue deformation characteristics [34].

The observed performance of 3D‐derived viscoelastic parameters suggests potential value for the characterization of early liver disease; however, these findings should be considered exploratory given the limited biopsy‐confirmed sample and marked class imbalance at F0. If confirmed in larger independent cohorts, earlier and more precise characterization of fibrotic changes could potentially support clinical risk stratification at disease stages in which lifestyle interventions may still have substantial benefit [35]. Likewise, emerging pharmacological therapies for MASH increase the need for accurate noninvasive phenotyping and staging, although the clinical utility of the present parameters for treatment selection remains to be established [36]. Wave attenuation and phase angle should therefore be regarded as surrogate biomechanical biomarkers whose biological determinants are likely multifactorial. Longitudinal studies are required to evaluate their evolution during disease progression and therapeutic response, whereas dedicated histopathological correlation with inflammatory activity, hepatocellular ballooning, perisinusoidal fibrosis, and other tissue characteristics may help clarify their biological basis [37]. Prospective longitudinal and histopathological validation in larger, stage‐balanced cohorts will be necessary before these parameters can be considered for clinical treatment selection or monitoring.

Several important limitations warrant careful consideration when interpreting our findings. First, the histopathological reference standard was available in 35 patients from our total cohort, despite rigorous control over biopsy timing and the strict blinding of all analyses to clinical outcomes. This reduces statistical power and may introduce selection bias because patients undergoing biopsy may not be representative of the overall MASLD/MASH population. In particular, the biopsy cohort contained only one F0 participant, making estimates for F0 classification highly sensitive to a single observation and precluding definitive conclusions regarding the detection of fibrosis‐free liver. Second, the limited number of biopsy‐confirmed participants reduces the precision of diagnostic‐performance estimates and increases the risk of model instability despite internal cross‐validation. Comprehensive multi‐center, multi‐scanner validation studies remain necessary to establish the generalizability of our findings across diverse patient populations and technical platforms. Third, the incremental clinical value of the MRE‐derived phase angle and wave attenuation parameters requires prospective outcome validation, including systematic correlations with established clinical endpoints such as hepatic decompensation events or progression to cirrhosis. The single‐center design of our investigation may limit the broader generalizability of our findings, and the absence of longitudinal follow‐up data precludes assessment of parameter evolution over time. The potential for selection bias within the biopsy subset constitutes an additional methodological limitation that warrants acknowledgment. Moreover, the 3D viscoelastic parameters were available only with the GT‐MRE implementation, whereas AC‐MRE was acquired using a conventional 2D sequence and product reconstruction. Consequently, differences between 3D GT‐MRE and 2D AC‐MRE cannot be attributed specifically to the GT excitation mechanism and may also reflect differences in acquisition dimensionality, sequence design, spatial sampling, or reconstruction methodology. Because VCTE and MRE were not invariably performed on the same day, interval‐related changes in liver status cannot be completely excluded.

Cost considerations and infrastructure accessibility remain significant barriers to the widespread implementation of advanced MRE technology. However, as 3D‐MRE hardware becomes increasingly integrated into standard MRI platforms and the clinical demand for noninvasive precision diagnostics continues to escalate, GT‐MRE techniques, particularly those incorporating comprehensive biomechanical modeling, may provide complementary biomechanical information beyond conventional stiffness measurement.

## Author Contributions

**Leon David Gruenewald:** conceptualization, methodology, investigation, data curation, formal analysis, validation, visualization, writing – original draft, writing – review and editing. **Christian Booz:** investigation, data curation, validation, writing – review and editing. **Jennifer Gotta:** investigation, data curation, writing – review and editing. **Philipp Reschke:** investigation, resources, writing – review and editing. **Tommaso D'Angelo:** investigation, visualization, writing – review and editing. **Nicole Suarez Ziegengeist:** data curation, validation, visualization. **Katerina Torgashov:** data curation, validation. **Laura Hempfling:** investigation, writing – review and editing. **Scherwin Mahmoudi:** investigation, resources. **Lisa Reisbeck:** data curation, validation. **Renate Maria Hammerstingl:** investigation, supervision, writing – review and editing. **Stefan Zeuzem:** resources, supervision. **Esra Görgülü:** investigation. **Anita Pathil‐Warth:** investigation. **Melis Onay:** investigation. **Eva Herrmann:** formal analysis, methodology, validation. **Iulia Dahmer:** formal analysis, validation. **Raja Idris:** investigation, resources. **Maria Johanna Gobertina Tetuanui Vehreschild:** resources, supervision. **Katharina Schregel:** investigation, writing – review and editing. **Christof Matthias Sommer:** supervision, writing – review and editing. **Sandra Ciesek:** resources, supervision. **Katrin Eichler:** supervision, writing – review and editing. **Thomas Joseph Vogl:** supervision, project administration, resources. **Ralph Sinkus:** conceptualization, methodology, software, supervision, writing – review and editing. **Sebastian Haberkorn:** software, methodology, writing – review and editing. **Vitali Koch:** conceptualization, methodology, formal analysis, funding acquisition, project administration, supervision, writing – original draft, writing – review and editing.

## Funding

- Those works performed under the RHU OPERANDI were supported in part by the French National Research Agency (Agence Nationale de la Recherche, ANR) as its 3rd PIA, integrated to France 2030 plan under reference ANR‐21‐RHUS‐0012. This work was supported by funding from the integrated cancer research center “SiRIC InsiTu: Insights into cancer: From inflammation to tumor” (grant number INCa‐DGOS‐INSERM‐ITMO Cancer_18008). This work was also supported by the French FHU MOSAIC2.
- Vitali Koch was funded by the ‘Bundesministerium für Forschung, Technologie und Raumfahrt’ (BMFTR, Federal Ministry of Research, Technology and Space)—01EO2102 INITIALISE Advanced Clinician Scientist Program. BMFTR funded the research rotation of Vitali Koch as a clinician scientist, but did not influence the content of the research. The funder had no role in study design, data collection and analysis, decision to publish, or preparation of the manuscript.
- Vitali Koch was funded by the “Doktor Robert Pfleger Foundation” (Bamberg, Germany).
- Vitali Koch was funded by the “Wilhelm Vaillant Foundation” (Munich, Germany).

## Ethics Statement

The institutional ethics review board approved this prospective study (Ethics Committee of Frankfurt am Main, Hesse, Germany; approval number 20‐887_2).

## Consent

Written informed consent was obtained from all participants prior to inclusion in the study. All figures and tables are original and created by the authors. No material from other sources requiring permission has been reproduced in this manuscript.

## Conflicts of Interest

Vitali Koch is a member of the RSNA and received travel support from Siemens Healthineers and Bayer. Christian Booz received speaking fees from Siemens Healthineers. Stefan Zeuzem reports consulting or speaker fees from AbbVie, Gilead, Intercept, Janssen, Merck/MSD, and Sobi. The other authors have no conflicts of interest to disclose. All authors have contributed significantly to this work to merit coauthorship and approved this manuscript and this submission. In particular, all authors took part in image analyses, statistical analyses, and scientific writing or editing.

## Supporting information


**Table S1:** Detailed study protocol of AC 2D‐MRE and GT 2D/3D‐MRE.

## Data Availability

Data generated or analyzed during the study are available from the corresponding author by request. All data associated with this study are present in the paper or the Supporting Information. Anonymized participant raw data regarding MRE and the necessary software to process the MRE data are available after signing a data access agreement with the host institution (University Hospital Frankfurt) and INSERM.
